# A variable probe pitch micro-Hall effect method

**DOI:** 10.3762/bjnano.9.192

**Published:** 2018-07-20

**Authors:** Maria-Louise Witthøft, Frederik W Østerberg, Janusz Bogdanowicz, Rong Lin, Henrik H Henrichsen, Ole Hansen, Dirch H Petersen

**Affiliations:** 1 DTU Nanotech, Technical University of Denmark, Building 345 East, DK-2800 Kgs. Lyngby, Denmark; 2 CAPRES A/S, Scion-DTU, Building 373, DK-2800 Kgs. Lyngby, Denmark; 3 IMEC, Kapeldreef 75, B-3001 Leuven, Belgium

**Keywords:** four-point probes, Hall effect, metrology, mobility, variable Probe Pitch

## Abstract

Hall effect metrology is important for a detailed characterization of the electronic properties of new materials for nanoscale electronics. The micro-Hall effect (MHE) method, based on micro four-point probes, enables a fast characterization of ultrathin films with minimal sample preparation. Here, we study in detail how the analysis of raw measurement data affects the accuracy of extracted key sample parameters, i.e., how the standard deviation on sheet resistance, carrier mobility and Hall sheet carrier density is affected by the data analysis used. We compare two methods, based primarily on either the sheet resistance signals or the Hall resistance signals, by theoretically analysing the effects of electrode position errors and electrical noise on the standard deviations. We verify the findings with a set of experimental data measured on an ultrashallow junction silicon sample. We find that in presence of significant electrical noise, lower standard deviation is always obtained when the geometrical analysis is based on the sheet resistance signals. The situation is more complicated when electrode position errors are dominant; in that case, the better method depends on the experimental conditions, i.e., the distance between the insulating boundary and the electrodes. Improvement to the accuracy of Hall Effect measurement results is crucial for nanoscale metrology, since surface scattering often leads to low carrier mobility.

## Introduction

Materials characterization becomes increasingly difficult as the dimensions of transistors continue to decrease. Although three dimensional electrical characterization is the ultimate goal of materials characterization, conventional metrology for thin-film characterization still plays an important role in development of materials used in nanoelectronics [1]. Hall effect measurements have been employed for decades to electrically characterize samples and extract important metrics, such as concentration, mobility and type of charge carriers [2–3]. Some of the measurement methods require significant sample preparation while other methods are destructive [2]. Great progress in measurement simplicity and accuracy has been achieved with the introduction of the micro-Hall effect (MHE) method [4]. The MHE measurement itself is performed simply by placing a micro four-point probe (M4PP) in parallel and close proximity to an insulating boundary, with an orthogonal magnetic field applied. Then the measured resistance will have three contributions: a drift term, a Hall effect term and a magnetoresitive term. In a comparative study by Clarysse et al., the MHE method has been shown to have higher accuracy than more conventional setups using square van der Pauw geometries [5]. Van der Pauw geometries often suffer from inaccurate contact placement, which easily results in measurement errors of a few percent [6]. Comparing the MHE method with measurements performed using a cloverleaf, Petersen et al. [7] have shown a 1:1 correlation between the measurements. Cloverleaf measurements are, however, challenging because of the sample definition required before any actual measurements can be performed. Hence, the MHE method holds several advantages over other well-known techniques, even though low-mobility samples can also be characterized by the latter [8].

The key to accurate extraction of sheet resistance *R*_0_, Hall sheet carrier density *N*_HS_ and Hall mobility μ_H_ from MHE measurements is to determine the exact distance between the probe and the insulating boundary. To this end, different measurement strategies have been described using micro four-point probes [4,9–11]. Most recently, a strategy based on variable probe pitch measurements using a multi-point probe with different subsets of four electrodes has been developed [11–12]. Similar strategies using variable probe pitch multi-point probes have been used for other systems, including current-in-plane tunneling measurements [13], junction-leakage measurements [14] and surface-conductivity measurements of bulk materials [15–16].

In this study, we present the variable probe pitch MHE method applied to an equidistant micro seven-point probe (M7PP), and compare two independent ways of extracting the relevant sample parameters from the same set of measurements. Furthermore, we will demonstrate the sensitivity of each method to position errors, as well as to electrical noise. Finally, we will present measurements on a B-doped Si ultrashallow junction, supporting our findings.

## Micro-Hall Effect Theory

**Figure 1:**
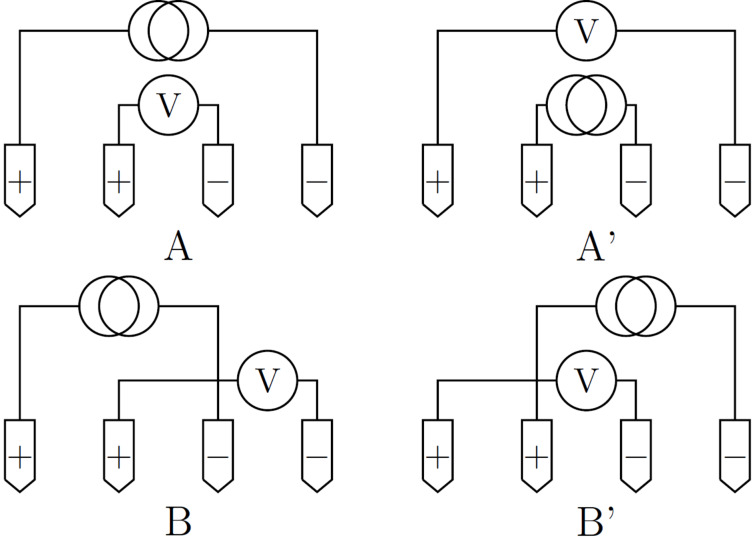
The standard probe pin configurations A, A’, B and B’ used in the experiments.

The fundamentals of Hall Effect measurements with a collinear M4PP have previously been described in detail [4]. However, we will briefly outline some of the most important characteristics here. For any four-point probe, 6 non-trivial configurations of current and electrode pins can be measured, but for this work, only the configuration pairs (A, A’) and (B, B’), illustrated in Figure 1, are relevant.

Crucial in understanding MHE measurements are the definitions of the resistance difference for the pairs, Δ*R*_XX′_ ≡ *R*_X_ − *R*_X′_, as well as their resistance average, 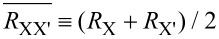, where 
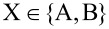
. For an equidistant four-point probe placed parallel to an insulating boundary, the resistance difference for the (B, B’) pair is

[1]
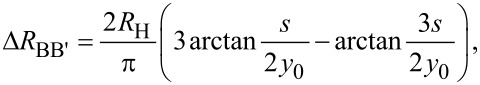


where *R*_H_ is the Hall sheet resistance, *s* is the electrode pitch and *y*_0_ the distance between the probe and the insulating boundary. Note that in the relevant case where the probe is placed parallel to a straight insulating boundary, the resistance difference for the (A, A’) pair is Δ*R*_AA′_ = 0. The resistance averages in the configuration pairs (A, A’) and (B, B’) are

[2]
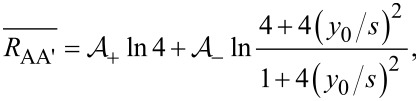


[3]
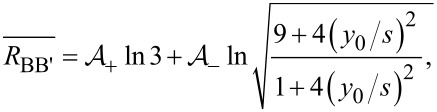


respectively, where the coefficients are given by

[4]
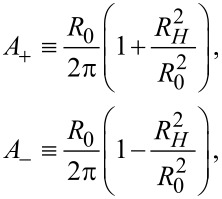


in which *R*_0_ denotes the sheet resistance. It has been shown that by introducing a pseudo sheet resistance *R*_p_, the effect of electrode position errors can be mitigated [9]. The pseudo sheet resistance is defined as the solution to the modified van der Pauw equation [17–20]

[5]
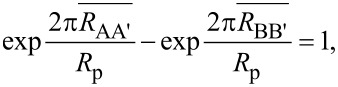


which will be utilized in the next section, in which the variable probe pitch method is presented in full.

## Variable Probe Pitch Method

**Figure 2:**
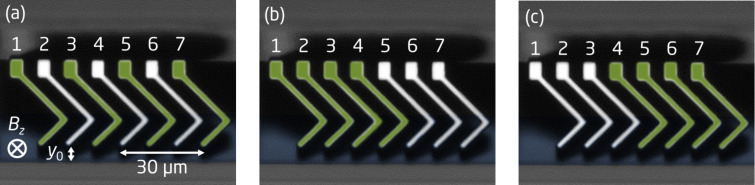
The three sub-probes on an M7PP used for multiplexing during measurements; a) 1357 (20 μm pitch), b) 1234 and c) 4567 (10 μm pitch). The electrode pins used in a given sub-probe are marked with green. The sample itself is highlighted in blue. The direction of magnetic flux density *B*_z_, which is pointing into the sample, is also indicated.

The variable probe pitch method uses measurements at different relative distances to the boundary of a sample by multiplexing several sets of four electrodes on a M7PP. These sets are called sub-probes and can be chosen with different electrode pitch. In this case, three equidistant sub-probes are used and named with reference to the index number of the four electrodes constituting the sub-probe, “1357” (20 μm pitch), “1234” and “4567” (10 μm pitch). The three sub-probes are outlined in Figure 2. Once resistance measurements have been performed in the A, A’, B and B’ configurations for the three sub-probes, two different ways of determining the distance to the boundary and ultimately obtaining the desired parameters, can be employed. The first method utilizes the Hall signal and will be referred to as the “Hall signal method”. The second method uses the resistance signal and will be referred to as the “resistance signal method”.

**Figure 3:**
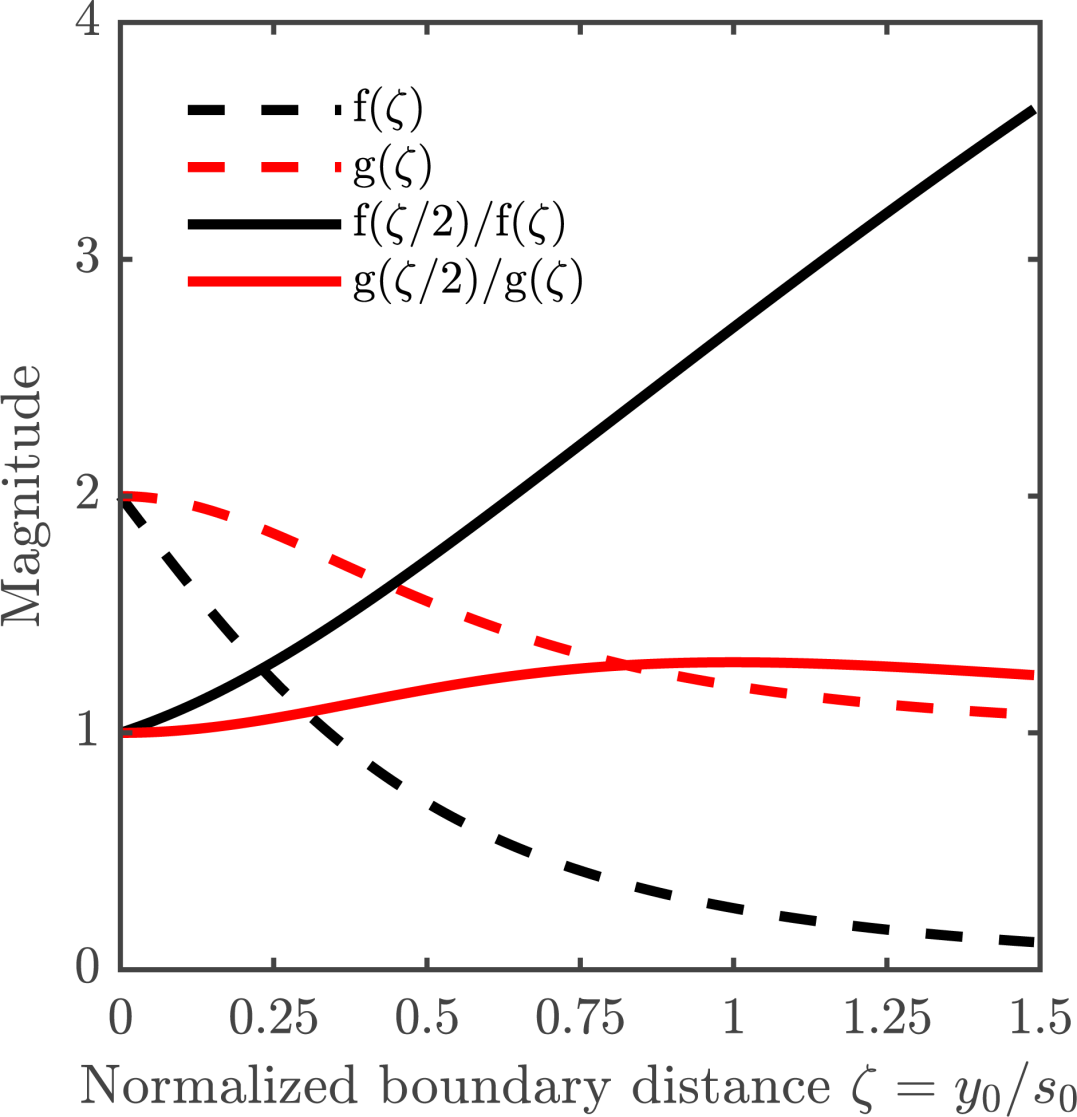
The dashed curves show the relative Hall signal Δ*R*_BB′_/*R*_H_ = f(ζ) (Equation 1) and relative pseudo sheet resistance *R*_P_/*R*_0_ = g(ζ) (Equation 5), as a function of normalized distance to the boundary ζ = *y*_0_/*s*_0_. The full curves show the ratios used in the Hall signal method Δ*R*_BB′2_/Δ*R*_BB′1_ = f(ζ/2)/f(ζ) and the resistance signal method *R*_P2_/Δ*R*_P1_ = g(ζ/2)/g(ζ), respectively, as a function of the normalized boundary distance.

To determine the distance to the boundary, *y*_0_, the first step in the Hall signal method is to exploit the fact that the Hall signal decreases with distance to the boundary relative to the probe pitch, as shown in Figure 3. In other words, it is possible to uniquely determine *y*_0_ by taking the ratio of two Hall signals (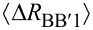 and Δ*R*_BB′2_) measured while using sub-probes with different pitches *s*_1_ and *s*_2_, i.e., by using the Hall signal Δ*R*_BB′2_ from the large probe, 1357, and the average of the Hall signals 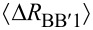 from the smaller probes, 1234 and 4567,

[6]
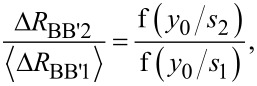


where f(·) is a geometrical function obtained from Equation 1.

To calculate *y*_0_ in the resistance signal method, dual-configuration position correction is utilized, by inserting the measured resistance averages for each sub-probe in the van der Pauw equation (Equation 5). From this equation, the pseudo sheet resistance is extracted, which, due to the presence of the boundary, differs from the true sheet resistance, *R*_0_. By measuring the pseudo sheet resistances, 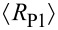 and *R*_P2_, at different relative distances to the boundary, using differently pitched (*s*_1_, *s*_2_) sub-probes, i.e., by using the resistance signal *R*_P2_ from the large probe, 1357, and the average of the resistance signals 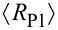 from the smaller probes, 1234 and 4567, it is possible to determine *y*_0_ from

[7]
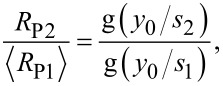


where g(·) is a geometrical function, implicitly found from Equation 2, Equation 3 and Equation 5.

Equation 6 and Equation 7 are plotted in Figure 3 for the specific case of a probe having the reference pitch *s*_0_ for the two smallest sub-probes and 2*s*_0_ for the larger sub-probe. They are plotted as a function of the normalized boundary distance ζ = *y*_0_/*s*_0_. We introduce *s*_0_ and ζ here to emphasize the nature and relationship between the sub-probes used in this paper; the pitches *s*_1_ and *s*_2_ utilized in this section are more general in nature and could also be used to describe other symmetric multipoint-probes.

After *y*_0_ has been calculated using either method, the Hall sheet resistance *R*_H_ and the sheet resistance *R*_0_ can be determined by means of

[8]
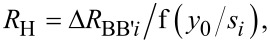


[9]
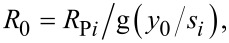


respectively, with 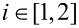. Finally, the Hall sheet carrier density, *N*_HS_, and the Hall mobility, μ_H_, can be found from [4]

[10]
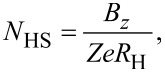


and

[11]
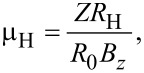


where *Z* is the sign of the charge carrier and *e* is the elementary charge.

**Figure 4:**
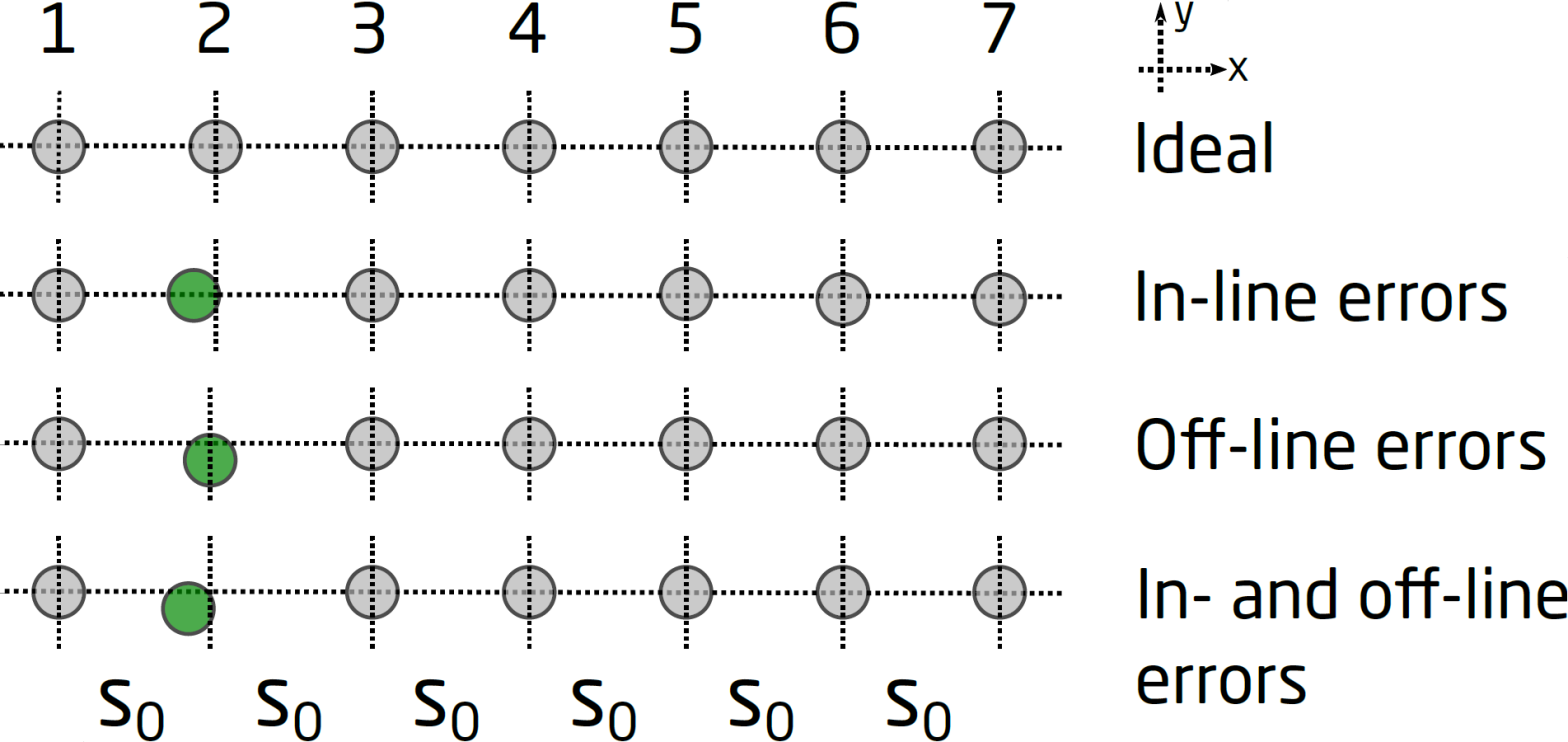
Static position errors: at the top, the ideal positions of a M7PP is shown. Below, the case of only in-line errors of the same probe can be seen, the affected pin marked with green (position error in the *x*-direction). Next, the case of only off-line errors is outlined using the same pin (position error in the *y*-direction) and at the bottom, a combination of in- and off-line errors on one of the pins is sketched.

The choice of using seven equidistant electrodes for the probe was carefully made, in order to minimize the contribution of in-line geometrical errors to the measured quantities, Δ*R*_BB′_ and *R*_P_. The definitions of in-line and off-line geometrical errors of an M7PP are illustrated in Figure 4. Any in-line errors on pins 1 and 7 will influence the size of the large sub-probe and the average size of the small sub-probes, which explains the correlation between values measured with sub-probe 1357, and the average values obtained with sub-probes 1234 and 4567. Furthermore, an in-line error on pin 4 would be inconsequential, as the pin is shared by the two 10 μm sub-probes. Such an error will cause an increase in the measured quantity of one sub-probe, whereas a decrease in the measured quantity will result from the other, leaving the average value unchanged. Hence, it should be possible to eliminate the correlated in-line errors on pins 1, 4 and 7, while in-line errors on pins 2, 3, 5 and 6 have low or zero influence on the measured quantities *R*_p_ and Δ*R*_BB′_. Off-line position errors can result in complex errors that are correlated to some extent, but these are beyond the scope of this study. Electrical noise will produce uncorrelated errors on the measurements, which cannot be corrected.

The sensitivity of the resistance signal method and the Hall signal method to position errors, as well as electrical noise, will be studied in the next section, to investigate which of the two independent methods perform best, and under which circumstances.

## Results and Discussion

In this section, we will evaluate numerically the expected measurement precision of the Hall signal method and the resistance signal method. The two main sources of error are geometrical errors and electrical noise, which we initially will discuss separately.

In the evaluation of geometrical errors, we will only consider mutually independent and normally distributed static position errors, meaning that if a position error is present on one of the electrode pins, this error will not change during a measurement. The relative standard deviation due to position errors, 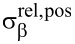, for a given property 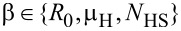 can be calculated from

**Figure 5:**
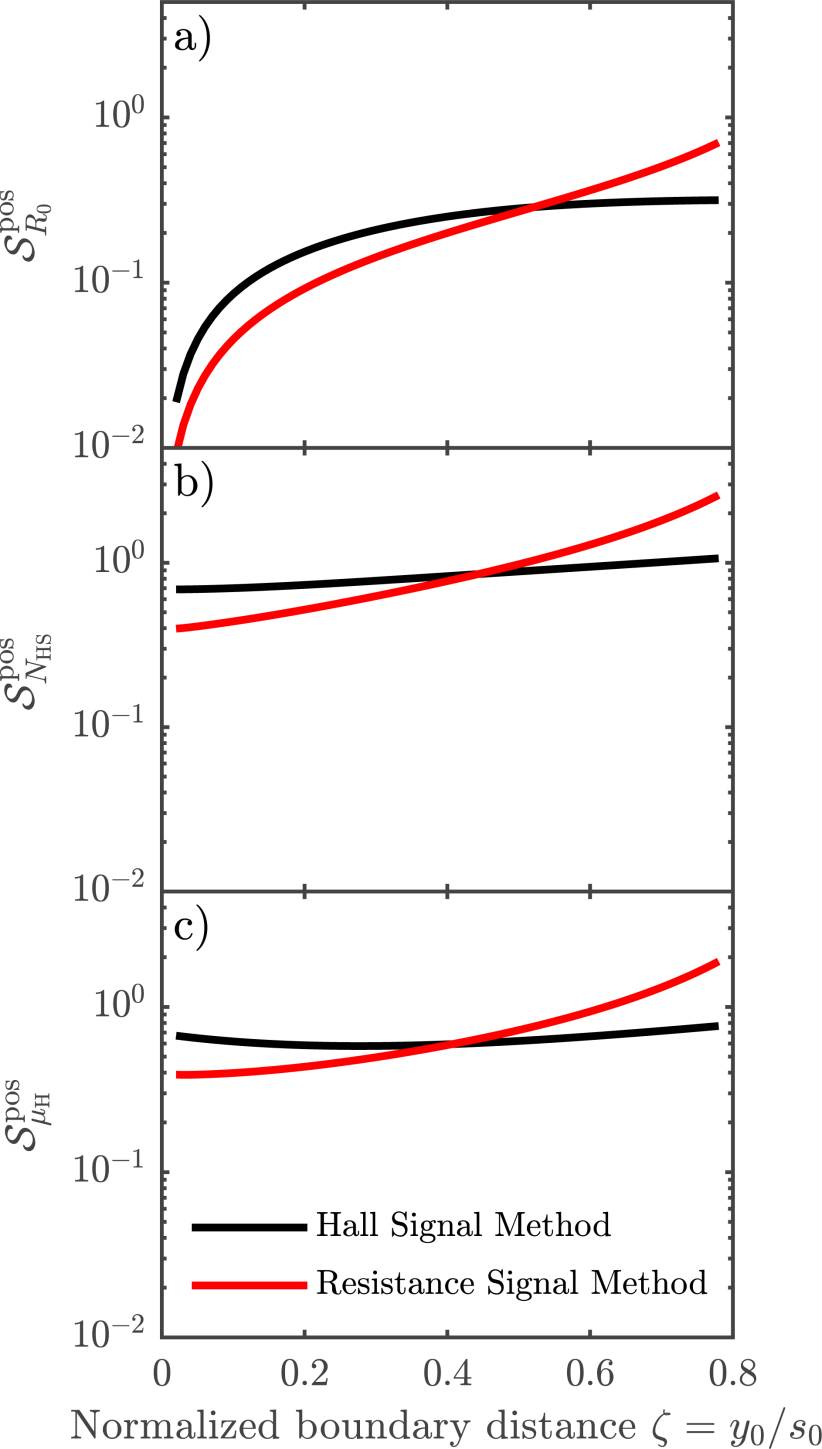
Effective sensitivity 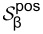 for a) *R*_0_, b) *N*_HS_ and c) μ_H_ when in- and off-line errors are present during the measurements. The resistance signal method results in the lowest sensitivities close to the edge, whereas the Hall signal method provides better results farther away from the boundary.

[12]
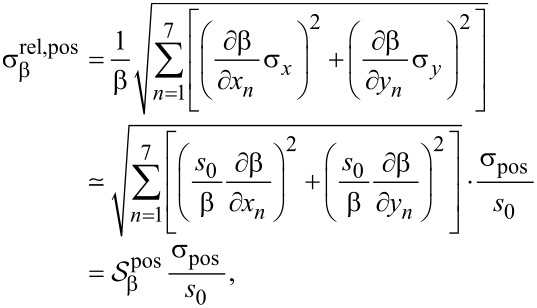


where we have normalized to the reference pitch *s*_0_. The standard deviations of in-line and off-line electrode position errors are assumed to be identical, σ_pos_ = σ*_x_* = σ*_y_*. The symbols *x_n_* and *y_n_* are the *x*- and *y*-positions, respectively, of the *n*-th electrode pin. 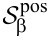 is the effective sensitivity of a given parameter to the relative position errors. This effective sensitivity is evaluated numerically for each parameter β and plotted in Figure 5, for both methods. The results generally predict an increased error with increasing distance from the boundary. The largest error is found for *N*_HS_ and the lowest for *R*_0_. Interestingly, the error of μ_H_ is lower than that of *N*_HS_, indicating a correlation of the errors of *R*_0_ and *R*_H_. This effect has previously been observed experimentally [9]. For the sheet resistance, the resistance signal method has the lowest relative standard deviation up to a distance of *y*_0_ = 0.53*s*_0_ away from the boundary, beyond which point a higher precision can be obtained using the Hall signal method. The same tendencies are displayed for the Hall mobility and the Hall sheet carrier density for which the method of highest accuracy changes at *y*_0_ = 0.41*s*_0_ and *y*_0_ = 0.45*s*_0_, respectively. The superiority of the resistance signal method closer to the boundary stems from the high precision on the pseudo sheet resistance ratio (Equation 7). For longer boundary distances, the pseudo sheet resistance ratio ceases to increase with boundary distance and finally starts declining as shown in Figure 3 and thus this ratio becomes less accurate for determining the boundary distance at larger distances. Since the resistance difference ratio (Equation 6) continues to increase with boundary distance, it becomes more suitable for determining the boundary distance and subsequently *R*_0_, *N*_HS_ and μ_H_ at larger boundary distances. Figure 3 also shows that the resistance signal method does not result in a unique solution for the boundary distance at larger boundary distances. Thus, it is necessary to place the probe within a distance of approximately *y*_0_ *< s*_0_ from the boundary.

To evaluate the contribution of electrical noise to MHE measurements, we consider twelve resistance measurements (*R_m_*, 
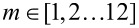
), i.e., four configurations for each sub-probe, in which a random voltage noise is present. The voltage noise comprises, e.g., Johnson noise from the two-point resistance and wiring resistance, as well as noise from the measurement electronics. The voltage noise is assumed to have the same standard deviation σ*_v_* = 60 nV for all twelve resistance measurements [21], which in turn are assumed to be uncorrelated. The voltage noise causes a noise in the resistance measurements with the standard deviation 
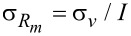
, where *I* is the measurement current. From the twelve configurations measured, the parameter β is calculated, and thus the relative standard deviation on β due to electrical noise is

**Figure 6:**
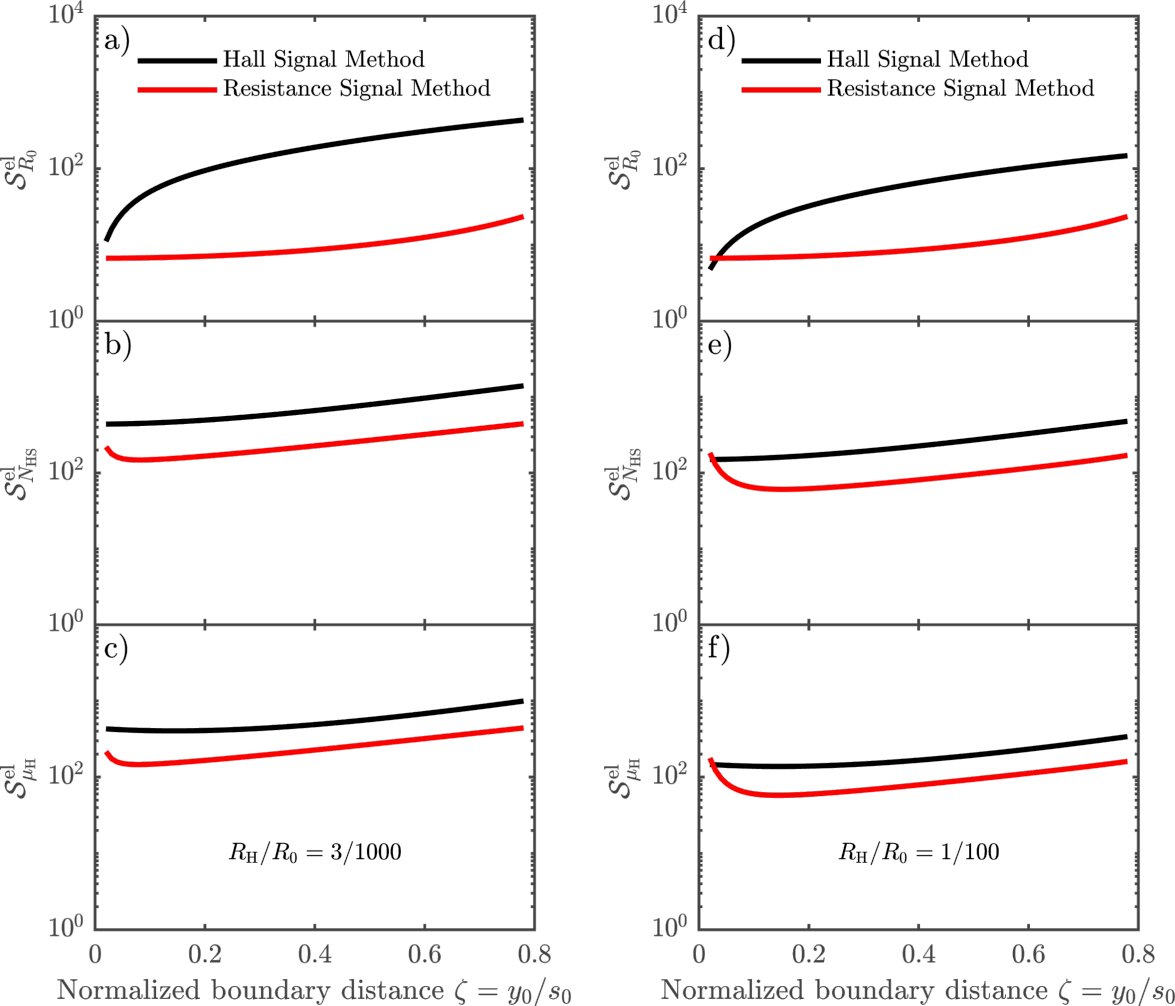
Effective sensitivity 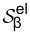 for the sheet resistance, Hall mobility and Hall sheet carrier density, due to the presence of electrical noise on the measurements, for the Hall signal method and the resistance signal method for (a–c) *R*_H_/*R*_0_ = 3/1000 and (d–f) *R*_H_/*R*_0_ = 1/100.

[13]
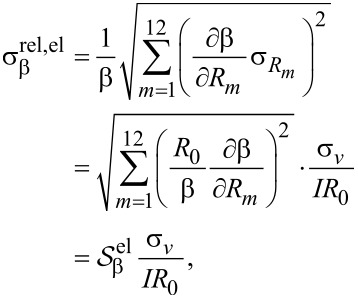


where 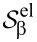 is a dimensionless sensitivity of β to electrical noise. This effective sensitivity has been calculated numerically for the sheet resistance, Hall mobility and Hall sheet carrier density, while choosing the ratio *R*_H_/*R*_0_ = 3:1000 to represent the experiments, and the ratio *R*_H_/*R*_0_ = 1:100 for comparison. These results are shown in Figure 6a–c and Figure 6d–f, respectively, for both methods. There are two main mechanisms describing the results shown in Figure 6: (1) the accuracy with which the distance to the boundary is determined and (2) how the position uncertainty translates into an error in the calculation of *R*_H_ and *R*_0_.

**Figure 7:**
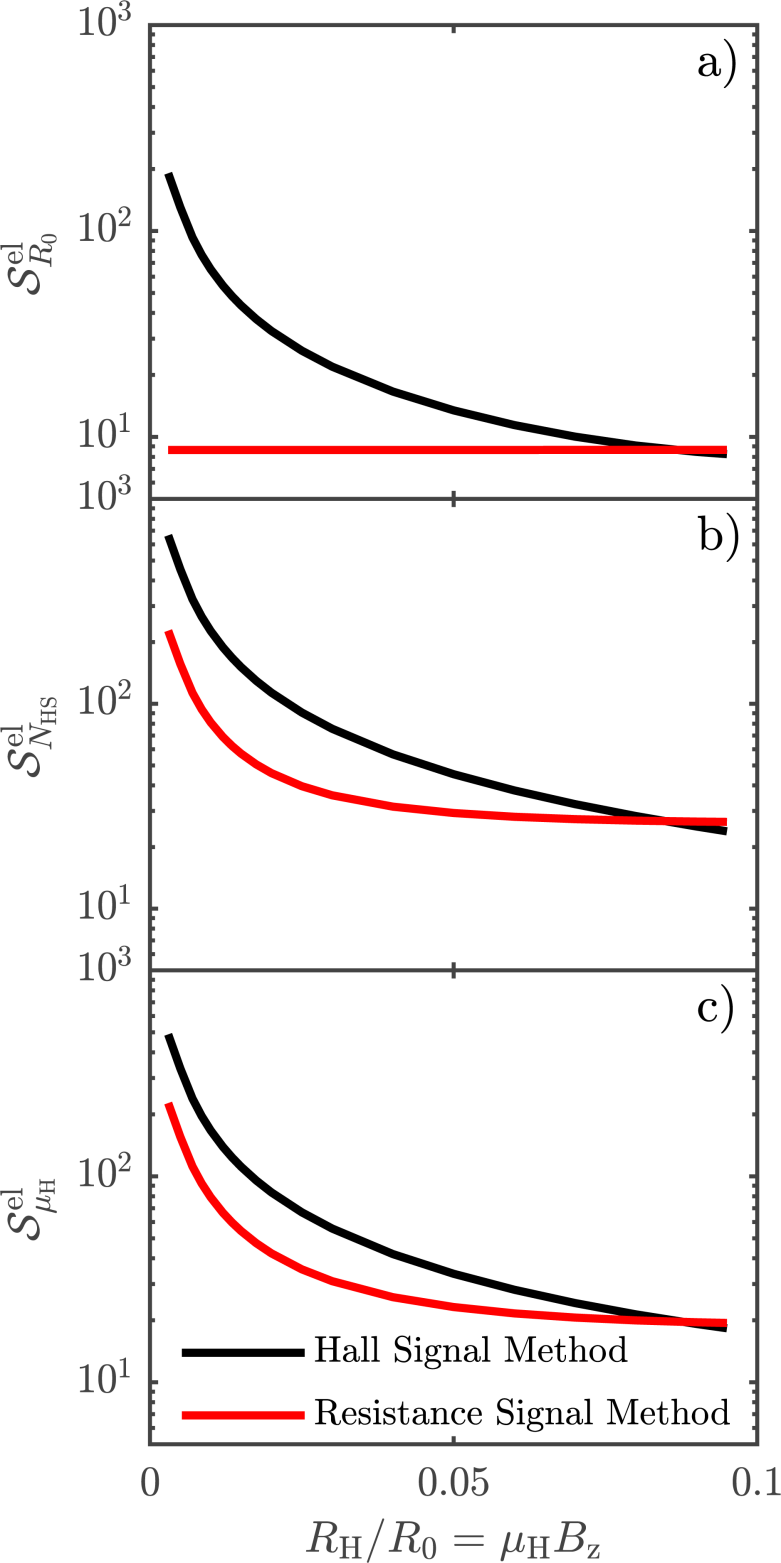
Generalization of Figure 6. The sensitivity of each parameter, a) Sheet resistance *R*_0_, b) Sheet carrier density *N*_HS_ and c) Hall mobility μ_H_, to electrical noise investigated for varying *R*_H_/*R*_0_ ratio, with the probe placed at a distance of *y*_0_ = 0.4*s*_0_ away from the insulating boundary.

Figure 7 generalizes the results from Figure 6 in the sense that the sensitivity of each parameter to electrical noise is investigated for varying *R*_H_/*R*_0_ ratios. A small *R*_H_/*R*_0_ ratio corresponds to a low mobility sample or a setup using a small magnetic field, whereas a higher ratio indicates the opposite. We have chosen to investigate *R*_H_/*R*_0_ ratios from 3 × 10^−3^ to 1 × 10^−1^, because of the nature of Equation 13, which takes into account only errors of first order. Investigating *R*_H_/*R*_0_ ratios below 3 × 10^−3^ would produce cases where the electrical noise we apply is comparable to or greater than the Hall signal, in which case Equation 13 is no longer valid. The probe is placed at a distance of *y*_0_ = 0.4*s*_0_ away from the insulating boundary, as it is most commonly done in experiments. Consider then the parameters *R*_0_, μ_H_ and *N*_HS_, as produced by the Hall signal method, and outlined in Figure 7a–c using black lines. In all three cases, we observe a similar relative decrease of 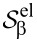 with increasing *R*_H_/*R*_0_ ratio. This makes sense, since the magnitude of the Hall signal Δ*R*_BB′_ compared to the resistance signal *R_m_* increases with the ratio *R*_H_/*R*_0_. Now consider the parameters as extracted from the resistance signal method, indicated by the red lines in Figure 7a–c. μ_H_ and *N*_HS_ display a similar behaviour as their counterparts in the Hall signal method, although with considerably lower magnitude errors, for the same reason as we have outlined previously. The error on *R*_0_ for the resistance signal method (Figure 7a) displays a behaviour radically different from the others. The reason is that *R*_0_ for the resistance signal method is determined completely without the influence of the Hall signal. Thus, a higher magnitude Hall signal will not result in a reduction of the uncertainty of *R*_0_. Instead, we see that the error of *R*_0_ is almost constant.

In a real measurement, both position errors and electrical noise are present, and thus the total relative standard deviation of β is

[14]
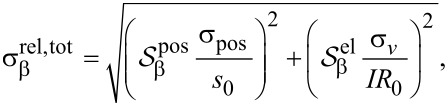


where either the first or the second term is dominant depending on the measurement conditions.

## Experimental

Measurements were performed using a microHall-A300 tool from CAPRES A/S and an M7PP with an electrode pitch of 10 μm. The M7PP used consisted of nickel-coated poly-silicon cantilever electrodes extending from the edge of a silicon die. A magnetic field with the flux density *B*_z_ = 600 mT was applied perpendicular to a boron-doped (10^15^ cm^−2^) shallow-junction Si sample. The probe was placed nominally 4 μm from the insulating boundary during measurements, i.e., *y*_0_/*s*_0_ = 0.2. A total of 150 engages was performed parallel to the insulating boundary, keeping the distance between the probe and insulating boundary constant. At each point, 75 configurations using A, A’, B and B’ configurations were measured; 25 for each of the sub-probes 1357, 1234 and 4567. The different parameters were then extracted using both the resistance signal method and the Hall signal method. The mean extracted values, as well as the standard deviations for each parameter are shown in Table 1.

**Table 1:** Mean values and standard deviations for the sheet resistance, Hall sheet carrier density and Hall mobility. The measurements were performed under 600 mT flux density and extracted using the resistance signal method and the Hall signal method.

Method	*R*_0_ ± Δ*R*_0_	*N*_HS_ ± Δ*N*_HS_	μ_H_ ± Δμ_H_
	Ω	10^14^ cm^−2^	cm^2^/(Vs)

Hall signal	284 ± 10	3.99 ± 0.34	56.8 ± 3.0
resistance signal	284 ± 2	3.95 ± 0.19	56.0 ± 2.4

Table 1 shows that the standard deviations for the resistance signal method are all lower than the corresponding standard deviations found for the Hall signal method. Based on the discussion about the sensitivities to both position errors and electrical noise, this meets the expectations. When a nominal distance to the edge of 4 μm is used during measurements, the resistance signal method should be the most accurate in all cases, according to Figure 5. Furthermore, it is observed that the largest relative deviations are found on the sheet carrier densities and the smallest on the sheet resistances, for both methods. This is also in line with our expectations. Finally, we find that the measurement results correspond to the case where the error is dominated by electrical noise.

## Conclusion

In this paper, we have presented a variable probe pitch method well-suited for characterization purposes in the development of nanoelectronic materials. We have compared two different analysis methods to obtain the electrical parameters *R*_0_, μ_H_ and *N*_HS_ from MHE measurement data. We have shown that the resistance signal method is more precise when measuring close to the insulating boundary of a sample, whereas the precision of the Hall signal method is better farther away from such a boundary, when static position errors are present. Furthermore, we have calculated the sensitivity of each method to electrical noise, and the resistance signal method proved superior. Finally, we presented MHE measurements on a B-doped Si ultra shallow junction and the experimental results confirmed the theoretical conclusions, since the standard deviations on the parameters were smaller for the resistance signal method, compared to those found for the Hall signal method.
